# Establishing Clinical and Laboratory Standards Institute M45 antimicrobial susceptibility testing methods and breakpoints for *Pseudomonas* other than *Pseudomonas aeruginosa*

**DOI:** 10.1128/jcm.00368-25

**Published:** 2025-06-30

**Authors:** Patricia J. Simner, Harley Harris, Emily Jacobs, Haley Stambaugh, Amira Bhalodi, Mark Fisher, Tsigereda Tekle, Jennifer Lu, Romney Humphries

**Affiliations:** 1 Division of Clinical Microbiology, Department of Laboratory Medicine and Pathology, Mayo Clinic 198422, Rochester, Minnesota, USA; 2 Division of Medical Microbiology, Department of Pathology, Johns Hopkins University School of Medicine 229385, Baltimore, Maryland, USA; 3 Scientific and Medical Affairs Consulting LLC, Newton, Pennsylvania, USA; 4 University of Utah and ARUP Laboratories 7060, Salt Lake City, Utah, USA; 5 Vanderbilt Medical Center University 12328, Nashville, Tennessee, USA; Endeavor Health, Evanston, Illinois, USA

**Keywords:** breakpoints, antimicrobial susceptibility testing, *Pseudomonas* species

## Abstract

The purpose of this study was to establish tentative Clinical and Laboratory Standards Institute (CLSI) M45 MIC and disk diffusion (DD) breakpoints (BPs) for *Pseudomonas* other than *Pseudomonas aeruginosa* (POPA). Mechanisms of antimicrobial resistance (AMR) and the modified carbapenem inactivation method (mCIM) to detect carbapenemase production were also evaluated. MIC data from *P. aeruginosa* and POPA from 2013 to 2022 were evaluated to compare the MIC distributions and modal MICs relative to the CLSI M100 *P. aeruginosa* BPs. A disk-to-MIC correlation study with 83 isolates was completed by testing reference broth microdilution and DD from the same inoculum, and the error-rate bounded method was used to establish DD BPs. For most antimicrobials, the modal MICs between *P. aeruginosa* and POPA were within 1-doubling dilution and lower than the M100 *P. aeruginosa-*susceptible BP. For amikacin, the modal MIC for POPA was 2-doubling dilutions lower than *P. aeruginosa* and was evaluated relative to the Enterobacterales BP. For aztreonam and trimethoprim-sulfamethoxazole, the modal MICs were elevated, and no BPs were set. New DD correlates were established for most antimicrobial agents, except for fluoroquinolones, where the *P. aeruginosa* correlates were suitable. AMR genes conferring resistance to multiple antimicrobial classes were identified by WGS. Beta-lactamase genes were identified in 30 (36.1%) isolates, with metallo-beta-lactamases (90.6%) predominating. The mCIM had a sensitivity and specificity of 100%. Upon review, the CLSI M45 committee proposed tentative MIC and DD BPs for expanded-spectrum cephalosporins (ceftazidime and cefepime), carbapenems (meropenem and imipenem), fluoroquinolones (ciprofloxacin and levofloxacin), and the aminoglycosides (amikacin and tobramycin).

**IMPORTANCE:**

*Pseudomonas* species other than *Pseudomonas aeruginosa* (POPA) can cause opportunistic infections which may be difficult to treat due to a variety of antimicrobial resistance mechanisms. Antimicrobial susceptibility testing is a critical component of patient management for these infections. Currently, the Clinical and Laboratory Standards Institute (CLSI) M100 non-Enterobacterales breakpoints and methodology are utilized for POPA by US clinical laboratories and likely do not accurately predict susceptibility results. The purpose of this study was to establish tentative CLSI M45 MIC and disk diffusion breakpoints for POPA. Mechanisms of antimicrobial resistance and the modified carbapenem inactivation method to detect carbapenemase production were also evaluated. We present the data used by the volunteers tasked by CLSI to develop POPA breakpoints in the M45 guidelines.

## INTRODUCTION

*Pseudomonas* is a complex and large genus of mostly aerobic, glucose-non-fermenting gram-negative bacteria that includes species with both clinical and environmental implications. With >250 validly published species, there are 12 species of primary clinical relevance to humans (1), 11 of which are *Pseudomonas* non-*aeruginosa* species (POPA). POPA are opportunistic pathogens affecting mostly immunocompromised hosts that have a predilection for the respiratory tract. Similar to *P. aeruginosa*, they are important airway colonizers and potential pathogens of patients with cystic fibrosis (2). *Pseudomonas putida, P. stutzeri* (a synonym of *Stutzerimonas stutzeri*), *and P. fluorescens* are the most common species recovered from clinical samples, encompassing ~90% of POPA isolated from clinical cultures (1, 3, 4).

POPA are intrinsically resistant to several beta-lactam agents (ampicillin, amoxicillin, amoxicillin-clavulanate, ampicillin-sulbactam, cefazolin, cefoxitin, cefotetan, cefuroxime, ceftriaxone, and ertapenem) and may develop multidrug-resistant phenotypes that contribute to difficulty in effectively managing infections caused by these pathogens. As such, antimicrobial susceptibility testing (AST) is required to guide successful treatment of infections. Furthermore, the acquisition of carbapenemases, more specifically metallo-β-lactamases (MBLs), among these organisms indicates its role as a potential reservoir and contributor to further spread of carbapenem-resistance among gram-negative organisms if left undetected (2, 5).

Currently, the CLSI non-Enterobacterales (nE) breakpoints (BPs) are used to interpret AST results for non-fastidious, glucose non-fermenting gram-negative bacilli without specific BPs, including POPA (6–8). There are numerous limitations to using the CLSI nE BPs, including the following: (i) these breakpoints have not been revised since their initial publication, (ii) only minimal inhibitory concentration (MIC) BPs are available, and (iii) agents with BPs in the table may be inappropriate for treatment of POPA infections (e.g., trimethoprim-sulfamethoxazole) (9). Furthermore, the United States Food and Drug Administration (FDA) does not recognize any CLSI nE BPs. Thus, most U.S. laboratories must rely on laboratory-developed tests when performing AST for POPA clinical management. Data supporting the use of commercial or automated AST systems for POPA are limited (1). Altogether, the limitations of available BPs and AST methodologies present significant uncertainty with regard to the clinical validity of AST results for POPA and raise patient safety concerns. In contrast to the CLSI, The European Committee on Antimicrobial Susceptibility Testing (EUCAST) has established BPs for *Pseudomonas* species, which can be applied to both *P. aeruginosa* and non-*aeruginosa* species, with the exception of novel beta-lactam combination agents and meropenem meningitis BPs that are limited to *P. aeruginosa* (10).

The primary objective of this study was to establish tentative MIC and disk diffusion breakpoints for POPA for inclusion in the forthcoming 4th Edition of the CLSI M45 document (7). The secondary objective was to determine genomic correlates of antimicrobial resistance (AMR) and to evaluate the modified carbapenem inactivation method (mCIM) for detection of carbapenemase production among POPA.

## MATERIALS AND METHODS

### Establishment of CLSI M45 MIC breakpoints

The process to establish BPs for the CLSI M45 guideline is less stringent than from the M100 standard that follows M23 guideline (11–13). *Pseudomonas* species MIC distribution data generated by reference BMD from isolates collected between 2013 and 2022 from the SENTRY database were evaluated (Table 1). These data included up to 469 POPA and 22,554 *P*. *aeruginosa* isolates for various antimicrobial agents over the same time frame. The modal MIC (most commonly observed value), MIC_50_ (MIC value at which ≥ 50% of the isolates are inhibited), MIC_90_ (MIC value at which ≥ 90% of isolates are inhibited), and tentative epidemiological cutoff values (tECV; MIC value that separates wild-type from non-wild-type populations) for each antimicrobial agent were determined (14). The ECVs were considered tentative as not all M23 criteria were met to establish true ECVs (e.g., data from ≥3 laboratories and on-scale MICs) (11). As data available for pharmacokinetic (PK)/ pharmacodynamic (PD) and clinical outcomes are less for POPA, PK/PD was extrapolated from *P. aeruginosa* and evaluated relative to the MIC distributions. For amikacin, PK/PD was extrapolated from the M100 Enterobacterales breakpoints (8).

**TABLE 1:** MIC distributions for *Pseudomonas aeruginosa* and *Pseudomonas* species other than *P. aeruginosa* from 2013 to 2022^*a*^

Antimicrobial agent	Dilution (µg/mL)														Total	MIC_50_	MIC_90_
	0.015	0.03	0.06	0.12	0.25	0.5	1	2	4	8	16	32	64	>			
Tobramycin (PA)				561	2,228	**10,987**	5,298	1,064	342	223				1,849	22,552	0.5	4
Tobramycin (POPA)				57	**217**	117*	16	7	3	6				15	438	0.25	1
Pip-tazo (PA)						733	338	1,325	**9,844**	3,203	2,161	1,355	1,039	2,545	22,543	4	>64
Pip-tazo (POPA)						20	38	38	95	**132**	98	22	11*	15	469	8	32
Imipenem (PA)				245	683	5,736	**8,841**	1,581	877	1,717				2,863	22,543	1	>8
Imipenem (POPA)				36	102	**177**	59	25*	24	18				27	468	0.5	4
Meropenem (PA)	125	262	1,287	2,432	**4,716**	4,224	2,844	1,572	1,299	1,224	1,242	667		645	22,539	0.5	16
Meropenem (POPA)			57	19	18	19	50	**135**	100	38				31	467	2	8
Cefepime (PA)				59	145	393	3,573	**7,978**	3,231	3,419	2,183			1,557	22,538	2	16
Cefepime (POPA)						91	54	**151**	111	24	12*			25	468	2	8
Ceftazidime (PA)	0	1	3	13	84	386	4,117	**9,383**	2,963	1,501	1,000	1,283		1,805	22,539	2	32
Ceftazidime (POPA)					48	25	66	**155**	92	19	11*			21	437	2	8
Aztreonam (PA)		3	10	82	381	446	299	906	**7,374**	6,250	2,581			4,202	22,534	8	>16
Aztreonam (POPA)				5	11	10	9	14	14	42	**120**			212	437	16	>16
Ciprofloxacin (PA)		562	2,160	**9,026**	2,890	2,146	1,256	971	747					2,762	22,520	0.12	>4
Ciprofloxacin (POPA)		76	75	**150**	78	23*	13	5	12					36	468	0.12	4
Levofloxacin (PA)		131	193	437	2,363	**9,328**	2,591	2,080	1,482					3,910	22,515	0.5	>4
Levofloxacin (POPA)				100	51	**143**	100	20	10*					43	467	0.5	4
Amikacin (PA)					113	443	742	4,821	**9,977**	3,690	1,149	501			22,549	4	16
Amikacin (POPA)					27	98	**157**	110	24*	9	3	0		10	438	1	4
SXT (PA)						808	618	3,481	7,851					**9,781**	22,539	4	>4
SXT (POPA)						87	35	56	72					**218**	468	4	>4

^*a*^ Pip-tazo: piperacillin-tazobactam; SXT: trimethoprim-sulfamethoxazole. Modal MIC is bolded, and epidemiological cutoff values (ECVs) are indicated by asterisks (*). PA, *Pseudomonas aeruginosa*; POPA, *Pseudomonas* species other than *P. aeruginosa*.

### Antimicrobial susceptibility testing and antimicrobial resistance testing

To establish disk correlates and evaluate mechanisms of AMR, 83 contemporary, geographically diverse isolates of POPA were collected from two U.S. clinical laboratories (Johns Hopkins University [JHU]; n: 12 and ARUP Laboratories; n: 20) and the U.S. Centers for Disease Control and Prevention (CDC, n: 51; Table 2) (13). To establish DD BPs, duplicate reference BMD and DD methods were performed in parallel from the same bacterial inoculum following CLSI guidelines (12, 13). For BMD, MHB (Difco, BD) was cation-adjusted, and antimicrobial powders were obtained from US Pharmacopeia (USP) and for DD, antibiotic disks (BD BBL Sensi-Disc, Sparks, MD) and MHA (Remel) were applied. For further details on the methods, see the methods described for the establishment of *Achromobacter* species breakpoints (9) as identical methods were applied for this study. QC was set up daily for both BMD and DD testing using *P. aeruginosa* ATCC 27853 and *Klebsiella pneumoniae* ATCC 700603.

**TABLE 2:** Summary of *Pseudomonas* species and beta-lactamase genes^*a*^

Organism ID	Number (%)	Beta-lactamase gene(s)
*Pseudomonas otitidis*	18 (21.7%)	*bla*_POM-1_ (17) and *bla*_POM-2_ (1)
*Pseudomonas* species	16 (19.3%)	*bla*_PAM-1_ (3), *bla*_PST-1_ (1), *bla*_VIM-2_ (1), *bla*_CARB-2_ & *bla*_DHA-1_ (1); *bla*_CARB-2_ & *bla*_IMP-13_ (1), and *bla*_PAM-2_ (1)
*Pseudomonas putida*	12 (14.5%)	
*Pseudomonas juntendi*	5 (6%)	
*Stutzerimonas stutzeri*	4 (4.8%)	*bla*_PST-2_ (1)
*Pseudomonas fluorescens*	3 (3.6%)	
*Pseudomonas fulva*	3 (3.6%)	
*Pseudomonas monteilii*	3 (3.6%)	
*Pseudomonas citronellolis*	2 (2.4%)	
*Pseudomonas kurunegalensis*	2 (2.4%)	
*Pseudomonas mosselii*	2 (2.4%)	
*Pseudomonas chengduensis*	1 (1.2%)	
*Pseudomonas koreensis*	1 (1.2%)	
*Pseudomonas mendocina*	1 (1.2%)	
*Pseudomonas moraviensis*	1 (1.2%)	
*Pseudomonas nitroreducens*	1 (1.2%)	
*Pseudomonas oryzihabitans*	1 (1.2%)	
*Pseudomonas plecoglossicida*	1 (1.2%)	
*Pseudomonas psychrotolerans*	1 (1.2%)	
*Pseudomonas qingdaonensis*	1 (1.2%)	
*Pseudomonas solani*	1 (1.2%)	*bla*_PAM-1_ (1)
*Pseudomonas soli*	1 (1.2%)	
*Pseudomonas tohonis*	1 (1.2%)	*bla*_PAM-3_ (1)
*Stutzerimonas* species	1 (1.2%)	
Total	83	

^*a*^ *bla*_POM_ (*P*. *otitidis* MBL), *bla*_PAM_ (*P. alcaligenes* MBL), *bla*_PST_ (subclass B1 metallo-beta-lactamase), *bla*_VIM_ (verona-integron MBL), *bla*_IMP_ (imipenemase), *bla*_CARB_ (carbenicillin-hydrolyzing class A beta-lactamase), and *bla*_DHA_ (class C cephalosporinase; first isolated in Dharan, Saudi Arabia).

### Establishing disk to MIC correlates

Using the AST data generated from the 83 POPA isolates, a scattergram was created for each antimicrobial to visualize the DD to MIC correlates. Using the tentative MIC BPs as described above, the error-bounded method was used to select the zone diameter BPs and to calculate discrepancy rates between MIC and zone diameter test results, as described by CLSI. By the error-rate-bound method, increased error rates are allowed for isolates within essential agreement (i.e., ±1 dilution of the breakpoint). The diffusion Breakpoint Estimation Testing Software (dBETS) tool was applied to generate DD BPs (http://dbets.shinyapps.io/dBETS/). Categorical agreement (CA), major errors (ME), very major errors (VME), and minor errors (MI) were evaluated, as appropriate. Any ME and VME results were repeated to rule out random testing errors. Acceptable VME, ME, and MI error rates were <10%, <10%, and <40% for isolates in the intermediate (I) *±* 1 category (i.e., isolates with BMD MIC values within 1 doubling dilution of the intermediate BP), respectively (11). For isolates in the <I–2 or >I+2 categories with BMD MIC values outside 1-doubling dilution of the SDD BP, and acceptable VME, ME, and MI error rates were <2%, <2%, and <5%, respectively (13).

### Carbapenemase detection

To evaluate the modified carbapenem inactivation method (mCIM) for detection of carbapenemase production among POPA, the mCIM was performed using a 10 uL inoculum from the purity plate inoculated as part of the AST study (8, 15, 16).

### Whole-genome sequencing (WGS)

Using the same bacterial inoculum that was used for the AST study, genomic DNA (gDNA) was extracted, and whole-genome sequencing was performed on the Illumina NextSeq platform and analyzed using the CDC PHoeNIx pipeline (17). All isolates were sequenced and analyzed at JHU, as previously described (9).

## RESULTS

### Tentative MIC breakpoints

The MIC distributions for each antimicrobial agent for POPA and *P. aeruginosa* are outlined in Table 1 and Supplemental Materials. For all antimicrobials except amikacin, aztreonam, trimethoprim-sulfamethoxazole, and meropenem, the modal MICs between *P. aeruginosa* and POPA were within 1-doubling dilution of each other and lower than the M100 *P. aeruginosa* susceptible BP. Meropenem had a 3-doubling dilution higher modal MIC for POPA compared to *P. aeruginosa*. However, applying the meropenem BPs for *P. aeruginosa*, we found the POPA modal MIC, and the majority of isolates were within the susceptible range, and thus we adopted these BPs for POPA as well. Aztreonam and trimethoprim-sulfamethoxazole presented an interesting distribution, with most isolates falling at MIC values ≥ 16 µg/mL or >4 µg/mL, respectively. Because of this distribution, breakpoints for aztreonam and trimethoprim-sulfamethoxazole were not established for POPA. For amikacin and POPA, the modal MICs (1 µg/mL) were 2-doubling dilutions lower than those of *P. aeruginosa* (4 µg/mL). For amikacin, *Enterobacterales* breakpoints were considered and deemed appropriate based on the amikacin MIC distribution of POPA. The proposed MIC BPs are summarized in Table 3.

**TABLE 3:** Tentative CLSI M45 4th Edition breakpoints for *Pseudomonas* species other than *P. aeruginosa*

Antimicrobial agent	MIC breakpoints^*a*^			Disk diffusion breakpoints		
	S	I	R	S	I	R
Piperacillin-tazobactam	≤16/4	32/4	≥64/4	≥22	17–21	≤16
Ceftazidime	≤8	16	≥32	≥19	15–18	≤14
Cefepime	≤8	16	≥32	≥19	14–18	≤13
Imipenem	≤2	4	≥8	≥20	15–19	≤14
Meropenem	≤2	4	≥8	≥18	14–17	≤13
Tobramycin	≤1	2	≥4	≥21	15–20	≤14
Ciprofloxacin	≤0.5	1	≥2	≥25	19–24	≤18
Levofloxacin	≤1	2	≥4	≥22	15–21	≤14
Amikacin	≤4	8	≥16	≥23	19–22	≤18

^*a*^ Interpretive criteria are adapted from those for *Pseudomonas aeruginosa*, as published in CLSI M100 in conjunction with evaluation of MIC distributions from surveillance data/clinical laboratories and PK/PD properties of the individual agent, with the exception of amikacin. For amikacin, *Enterobacterales* systemic breakpoints were considered and deemed appropriate based on the amikacin MIC distribution of non-*aeruginosa Pseudomonas*.

### Disk diffusion correlates

To provide an additional AST method for use in clinical laboratories, we correlated the BMD results to disk diffusion measurements to establish disk diffusion breakpoints. Using the tentative MIC breakpoints and the dBETS program, the CLSI M45 committee established the disk diffusion breakpoints (Table 3; Supplemental materials). The disk diffusion correlates to BMD MIC results are summarized in Table 4. For fluoroquinolones, the correlates matched well to *P. aeruginosa* disk breakpoints of susceptible (S)/I/resistant (R) ≥25/19–24/≤18 mm for ciprofloxacin and ≥22/15–21/≤14 mm for levofloxacin and were set accordingly. For ciprofloxacin, applying *P. aeruginosa* disk correlates resulted in a higher I+1 and I–1 mE rate at 57%, but the M45 working group found this acceptable and favored harmonizing with the *P. aeruginosa* disk BPs. However, for the other drugs, the small size of the intermediate range from the *P. aeruginosa* BPs did not meet current M23 criteria (11). For these cases, we re-evaluated the disk correlates with wider intermediate ranges using the error-rate bounded method. For the cephalosporins, BPs were set to ≥19/15–18/≤14 mm for ceftazidime and ≥19/14–18/≤13 mm for cefepime. Piperacillin-tazobactam (TZP) BPs were set at ≥22/17–21/≤16 mm. BPs for imipenem were established at ≥20/15–19/≤14 mm to meet M23 criteria. For tobramycin, most of the isolates tested as susceptible with very few resistant isolates and no intermediate results, which made evaluating BPs challenging. The *P. aeruginosa* BPs would have set a very small resistance category zone of ≤12 mm. To adjust the intermediate zone to meet M23 guidelines and expand the resistance range, BPs were set to ≥21/15–20/≤14. Meropenem correlates did not match *P. aeruginosa* disk breakpoints. As discussed previously, the MIC distributions are different for POPA vs *P. aeruginosa*, so it was deemed reasonable to define different disk BPs for meropenem with POPA. Thus, the breakpoints were set to ≥18/14–17 /≤13 mm. For amikacin, the disk BPs were set as ≥20/17–19/≤18 mm based on the DBETS software recommendations. All results were within acceptance criteria with a few exceptions where ≥I+2 piperacillin-tazobactam, imipenem, and tobramycin were elevated due to a few isolates and a small denominator. Thus, the M45 committee found these small deviations acceptable.

**TABLE 4:** Disk diffusion correlates to reference broth microdilution minimal inhibitory concentration results^*a*^

Range	N	VME	ME	mE
Piperacillin/tazobactam				
≥I+2	5	0		1 (20%)
I+1 and I–1	29	0	0	7 (24.1%)
≤I–2	49		0	1 (2%)
Total	83	0	0	9 (10.8%)
Ceftazidime				
≥I+2	5	0		0
I+1 and I–1	19	0	0	5 (26.3%)
≤I–2	58		0	0
Total	82	0	0	5 (6.1%)
Cefepime				
≥I+2	2	0		0
I+1 and I–1	16	0	0	4 (25%)
≤I–2	64		0	0
Total	82	0	0	4 (4.9%)
Imipenem				
≥I+2	3	0		1 (33.3%)
I+1 and I–1	13	0	0	4 (30.7%)
≤I–2	67		0	0
Total	83	0	0	5 (6.0%)
Meropenem				
≥I+2	10	0		0
I+1 and I–1	48	0	0	17 (35.4%)
≤I–2	25		0	0
Total	83	0	0	17 (20.5%)
Tobramycin				
≥I+2	4	0		1 (25%)
I+1 and I–1	5	0	0	1 (20%)
≤I–2	74		0	0
Total	83	0	0	2 (2.4%)
Ciprofloxacin				
≥I+2	8	0		0
I+1 and I–1	14	0	0	8 (57.14%)
≤I–2	61		0	0
Total	83	0	0	8 (9.6%)
Levofloxacin				
≥I+2	10	0		0
I+1 and I–1	26	0	0	9 (34.6%)
≤I–2	47		0	2 (4.3%)
Total	83	0	0	11 (13.2%)
Amikacin				
≥I+2	0	0		0
I+1 and I–1	8	0	0	2 (25%)
≤I–2	75		0	0
Total	83	0	0	2 (2.4%)

^*a*^ Grey shading indicates that the result is not applicable.

### Whole-genome sequencing results

Whole-genome sequencing (WGS) was performed to confirm the species identification of the isolates and determine genetic correlates of resistance. The final breakdown of POPA species identified by WGS is summarized in Table 2. The most common species among isolates were *P. otitidis* (21.7%)*, P. putida* (14.5%)*, P. juntendi* (6%), and *S. stutzeri* (4.8%). Interestingly, many (n: 66; 81%) of the isolates submitted to the study had identifications of *Pseudomonas*, not *aeruginosa;* highlighting the inability to confidently achieve species-level identifications by clinical laboratories.

With the rise of carbapenem resistance among non-fermenters, particularly via MBL, and the possibility of POPA being a reservoir for resistance (18, 19), we looked for the presence of beta-lactamase genes in our sequenced isolates and if this correlated to phenotypic resistance in BMD results. Beta-lactamase genes (n: 32) were identified in 30 out of 83 isolates. Most beta-lactamase genes detected were MBLs, including 18 *bla*_POM_ (*P. otitidis* MBL) (20), six *bla*_PAM_ (*P*. *alcaligenes* MBL), two *bla*_PST_ (subclass B1 metallo-beta-lactamase), two *bla*_VIM_ (verona-integron MBL), and one *bla*_IMP_ (imipenemase). Only three of the beta-lactamases encountered had no carbapenem hydrolyzing activity, including two *bla*_CARB_ (carbenicillin-hydrolyzing class A beta-lactamase) and one *bla*_DHA_ (class C cephalosporinase, first isolated in Dharan, Saudi Arabia). Applying the tentative breakpoints, 44 (53%) and 4 (4.8%) of isolates were not susceptible (i.e., intermediate or resistant) to meropenem and imipenem, respectively. Despite the lower tentative carbapenem breakpoints as compared to the nE BPs that were previously applied for POPA, MBLs were identified among meropenem-susceptible (n: 7), -intermediate (n: 12), and -resistant (n: 10) isolates. For the remaining meropenem non-susceptible isolates where an MBL was not detected (n: 21), 11 (52.4%) harbored a multidrug-resistant efflux pump, including *ttgABC* operon (n: 9) or *tmexCD-toprJ* (n: 2) RND-type efflux pump gene cluster variants (21). Additional resistance genes to other classes were detected, including aminoglycoside resistance (*aph, aac, aad, and ant*), trimethoprim-sulfamethoxazole resistance (*sul1 and dfrA*), quaternary ammonium resistance (*qac*), chloramphenicol resistance (*cmlA1*), and fluoroquinolone resistance (*qnrA and crpP*).

### Modified carbapenem inactivation method results

The mCIM proved robust against POPA, demonstrating a sensitivity and specificity of 100% as compared to WGS. Importantly, it demonstrated positive results even when the carbapenems tested susceptible by the tentative breakpoints for isolates harboring MBL genes, five *bla*_PAM_ and two *bla*_POM_.

## DISCUSSION

In the United States, the CLSI nE BPs have historically been applied to POPA to interpret AST results to guide patient care. Due to the limitations of this approach, the CLSI M45 committee set out to develop AST methods and breakpoints for POPA. Ideally, the goal of the committee was to try to harmonize the breakpoints with those established for the closely related *P. aeruginosa,* as published in the CLSI M100 document.

The tentative MIC BPs for POPA that are summarized in this study and proposed by the M45 committee largely harmonize with those for *P. aeruginosa,* with a few exceptions. Those exceptions include amikacin, where the modal MIC was 2-dilutions lower for POPA when compared to *P. aeruginosa*. The aminoglycoside BPs were recently revisited for *P. aeruginosa,* where a urine-only breakpoint was established for amikacin. A systemic breakpoint was not favored due to the wild-type abutting the MIC typically achieved using standard dosing for this agent. However, due to the ability of amikacin to concentrate in the urine and clinical efficacy studies data demonstrating that outcomes are most reliable for infections originating in the urinary tract, a urine-only breakpoint was established. For POPA, it was decided to set a systemic breakpoint as the modal MIC was 2-doubling dilutions lower for POPA as compared to *P. aeruginosa*. As such, the Enterobacterales amikacin BPs were adopted, which fit the MIC distributions observed for POPA. Furthermore, the modal MIC for aztreonam was above the MICs typically achievable by routine antimicrobial dosing for *P. aeruginosa,* indicating limited activity of the antimicrobial agent (22). Similar to *P. aeruginosa*, POPA demonstrated elevated trimethoprim-sulfamethoxazole modal MICs. Thus, BPs were not set for aztreonam and trimethoprim-sulfamethoxazole for POPA.

Aligning the disk diffusion breakpoints with *P. aeruginosa* was more challenging as the historical disk correlates did not align with current M23 guidelines, as the intermediate range for many of the agents was too small. As such, new disk correlates were set for most agents with the exception of the fluoroquinolones, where *P. aeruginosa* breakpoints could also be applied to POPA and met M23 guidelines.

Many isolates submitted to the disk correlate study by clinical laboratories across the United States were identified as *Pseudomonas* non-*aeruginosa,* indicating difficulty by most laboratories to identify non-*aeruginosa* to the species level. The distribution of species based on whole-genome sequencing results was interesting. The predominant species included in this study was *P. otitidis*. However, that likely reflects study design rather than a predominance of *P. otitidis* infections as the isolates submitted by the CDC were enriched for carbapenem-resistant isolates to help with the evaluation of the mCIM in this study. All *P. otitidis* included in this study harbored an MBL *bla*_POM_ that is intrinsic and named after this species (20). The remaining distribution of isolates was to be expected, with *P. putida, S. stutzeri,* and *P. fluorescens* being among the most common species encountered. The list of most common species included *P. juntendi* (23), a species that was described in 2018 and isolated from a sputum sample of a patient in Japan. *P. juntendi* is part of the *P. putida* group and is closely related to *Pseudomonas asiatica*, *Pseudomonas monteilii,* and *P. putida*. It should also be noted that an additional 18 species were identified, highlighting the diversity of species encountered clinically. Even applying WGS, 16 isolates were identified to the genus level, highlighting the challenges encountered in identifying these bacteria in routine clinical labs. Due to these difficulties, the CLSI M45 committee added guidelines to the M45 document that if the identification of a *Pseudomonas* species isolate is uncertain, it is recommended that the *P. aeruginosa* breakpoints as outlined in the more frequently updated CLSI M100 document be applied as the most conservative approach.

Isolates in this study were enriched for carbapenem-resistant isolates, and as such, many isolates were found to harbor beta-lactamases, specifically MBLs or efflux pumps contributing to carbapenem resistance. The predominance of MBLs among POPA is concerning as there are limited therapeutic options available for MBL producers and highlights that POPA may serve as a reservoir for the further spread of MBLs, including acquired variants like *bla*_VIM_ and *bla*_IMP_. Importantly, despite lowering the carbapenem BP relative to the nE BPs, there were still 24% (7 of 29) of MBL-producers that tested susceptible to the carbapenems. Importantly, the mCIM performed very well among POPA with 100% sensitivity and specificity. As such, laboratories may consider validating and implementing the mCIM or another phenotypic carbapenemase assay prior to reporting the carbapenems to indicate the presence of a carbapenemase. A recent study from Brazil demonstrated good performance of various phenotypic tests (Carba NP, Blue Carba, and mCIM) for the detection of carbapenemase-producing *Pseudomonas* spp., including *P. putida* isolates (24).

There are some limitations to this study. First, the BPs developed are based almost entirely on MIC distribution data from a single source and lacked PK/PD and patient outcomes data (13). Another limitation is that the DD correlate study was conducted at a single center using a single lot and manufacturer of disks and media. While appropriate quality controls were used to ensure test the accuracy, the impact of site-to-site variability or manufacturer differences is not known.

In summary, we present the data used by the volunteers tasked by the CLSI to develop *Pseudomonas* other than *P. aeruginosa* species BPs in the M45 guideline. Upon review, the CLSI M45 committee proposed tentative MIC and DD BPs for expanded-spectrum cephalosporins (ceftazidime and cefepime), carbapenems (meropenem and imipenem), fluoroquinolones (ciprofloxacin and levofloxacin), and the aminoglycosides (amikacin and tobramycin). Laboratories may review the data presented here with their antimicrobial stewardship committee to adopt the proposed tentative clinical breakpoints, or alternatively, they can decide to wait until the official publication in the forthcoming 4th Edition of the CLSI M45 document in early 2026.

## Data Availability

The WGS data were deposited to the SRA under BioProject PRJNA1222910.

## SUPPLEMENTAL MATERIAL

Supplemental materials jcm.00368-25-s0001.pdf M45 POPA histograms and scattergrams.

ASM does not own the copyrights to Supplemental Material that may be linked to, or accessed through, an article. The authors have granted ASM a non-exclusive, world-wide license to publish the Supplemental Material files. Please contact the corresponding author directly for reuse.
