# Supplementary material for: Establishing Clinical and Laboratory Standards Institute M45 antimicrobial susceptibility testing methods and breakpoints for Pseudomonas other than Pseudomonas aeruginosa
Source: J Clin Microbiol. 2025 Jun 30;63(8):e00368-25. doi: 10.1128/jcm.00368-25 (PMC12345185; doi:10.1128/jcm.00368-25)

Pseudomonas species other than *P. aeruginosa* (POPA)

Amikacin MIC data (n = 438)

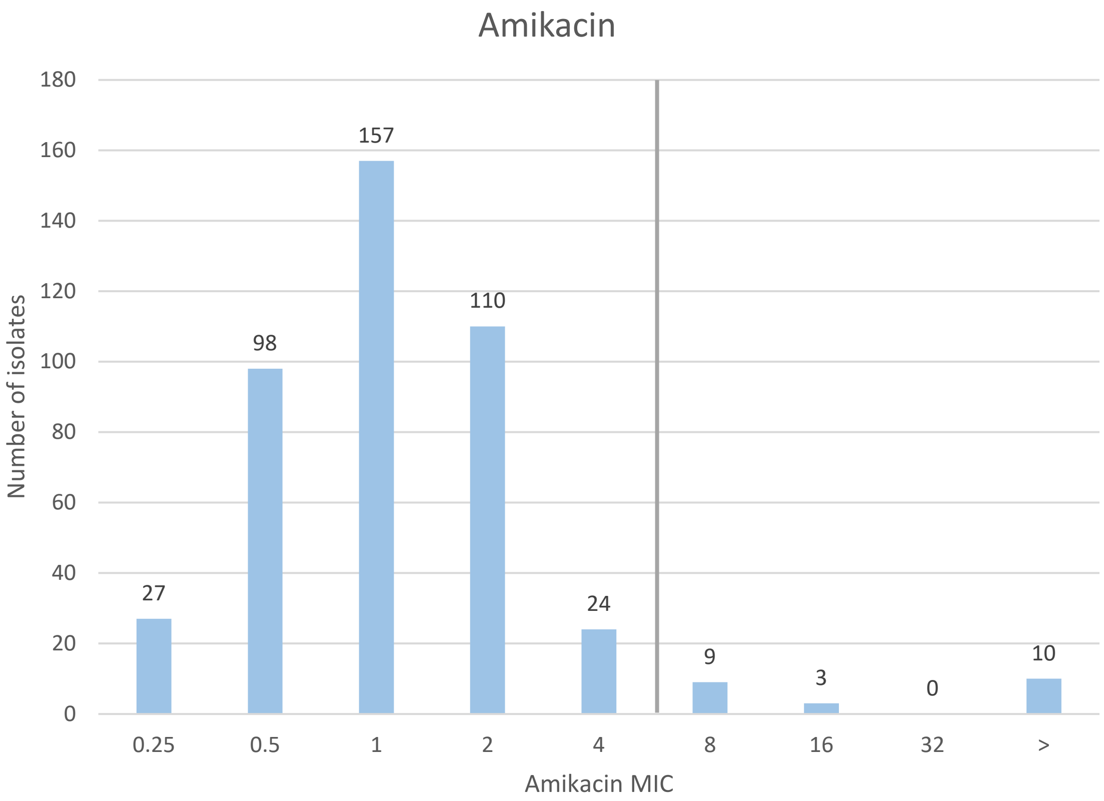

ECOFF Finder @ 97.5%: 4 ug/ml

**Proposed MIC breakpoints**		
**S**	**I**	**R**
<=4	8	>16

Aztreonam MIC data (n = 437)

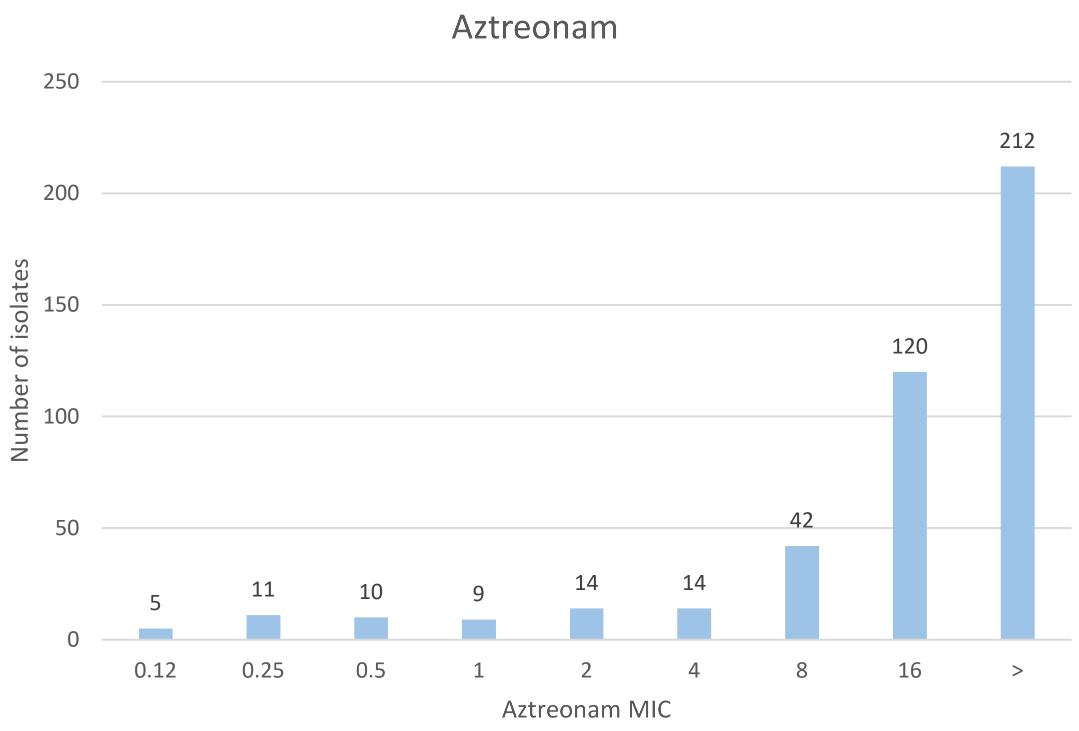

ECV could not be calculated

No proposed breakpoints

Cefepime MIC data (n = 468)

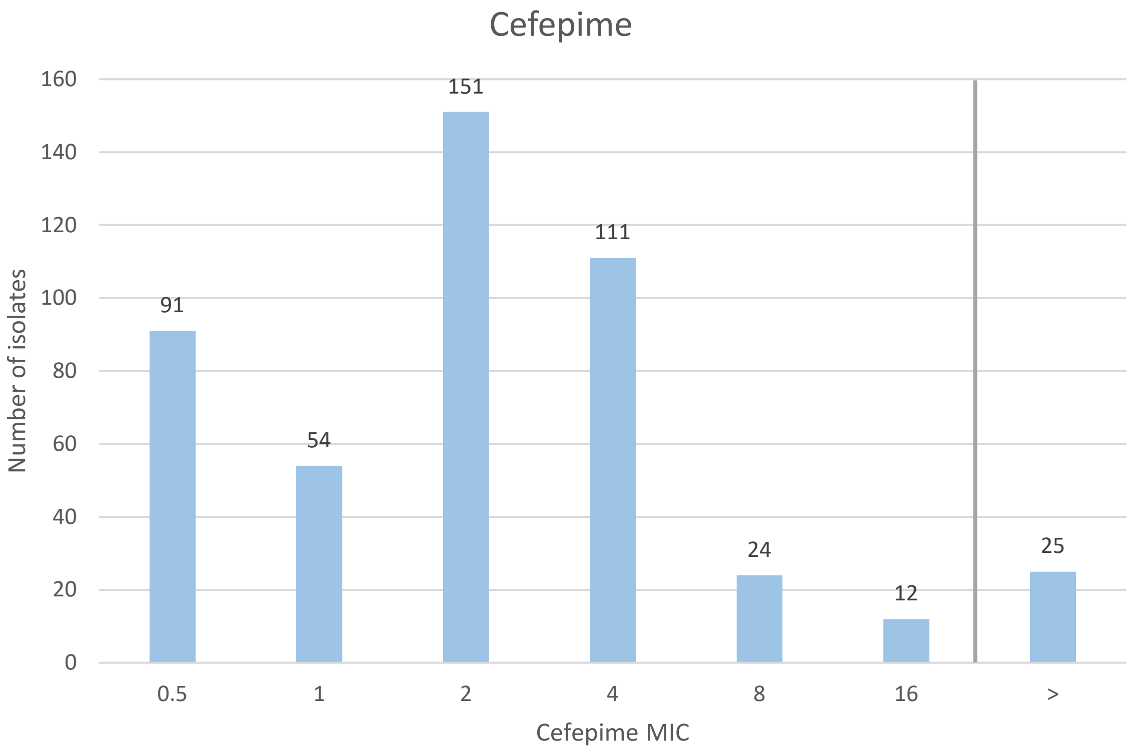

ECOFF Finder @ 97.5%: 16 ug/ml

Proposed MIC breakpoints		
**S**	**I**	**R**
<=8	16	>32

Ceftazidime MIC data (n = 437)

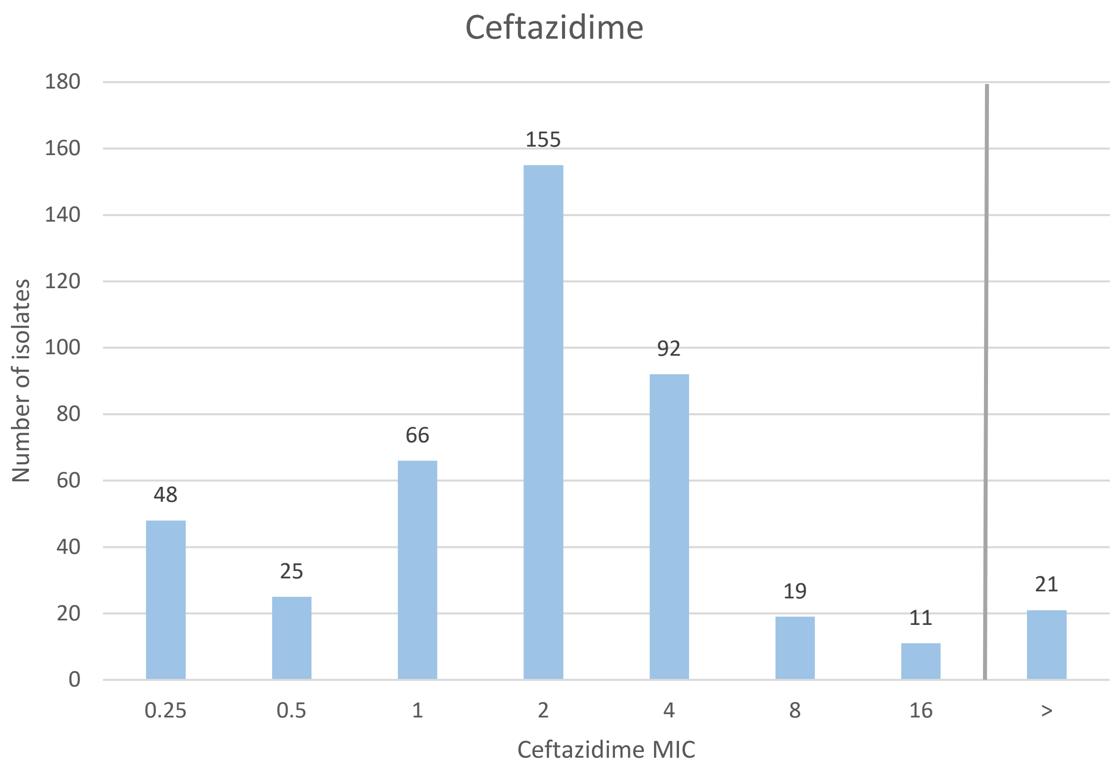

ECOFF Finder @ 97.5%: 16 ug/ml

**Proposed MIC breakpoints**		
**S**	**I**	**R**
<=8	16	>32

Ciprofloxacin MIC data (n = 468)

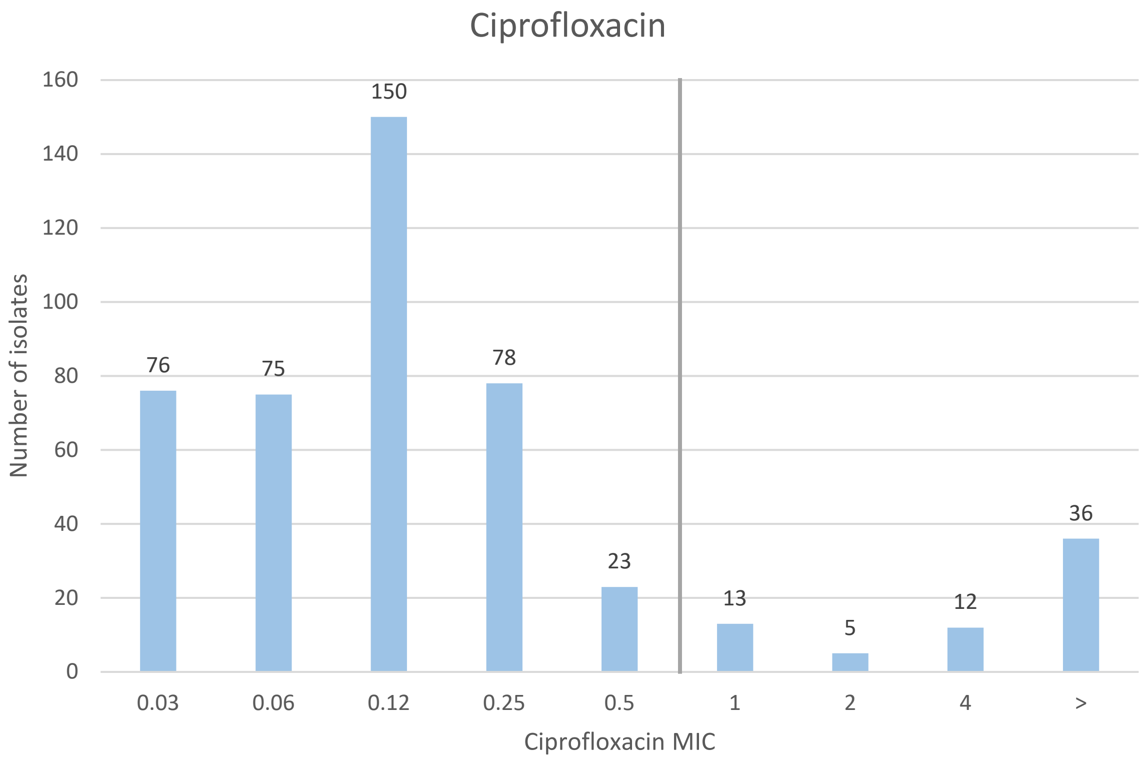

ECOFF Finder @ 97.5%: 0.5 ug/ml

Proposed MIC breakpoints		
**S**	**I**	**R**
<=0.5	1	>2

Imipenem MIC data (n = 468)

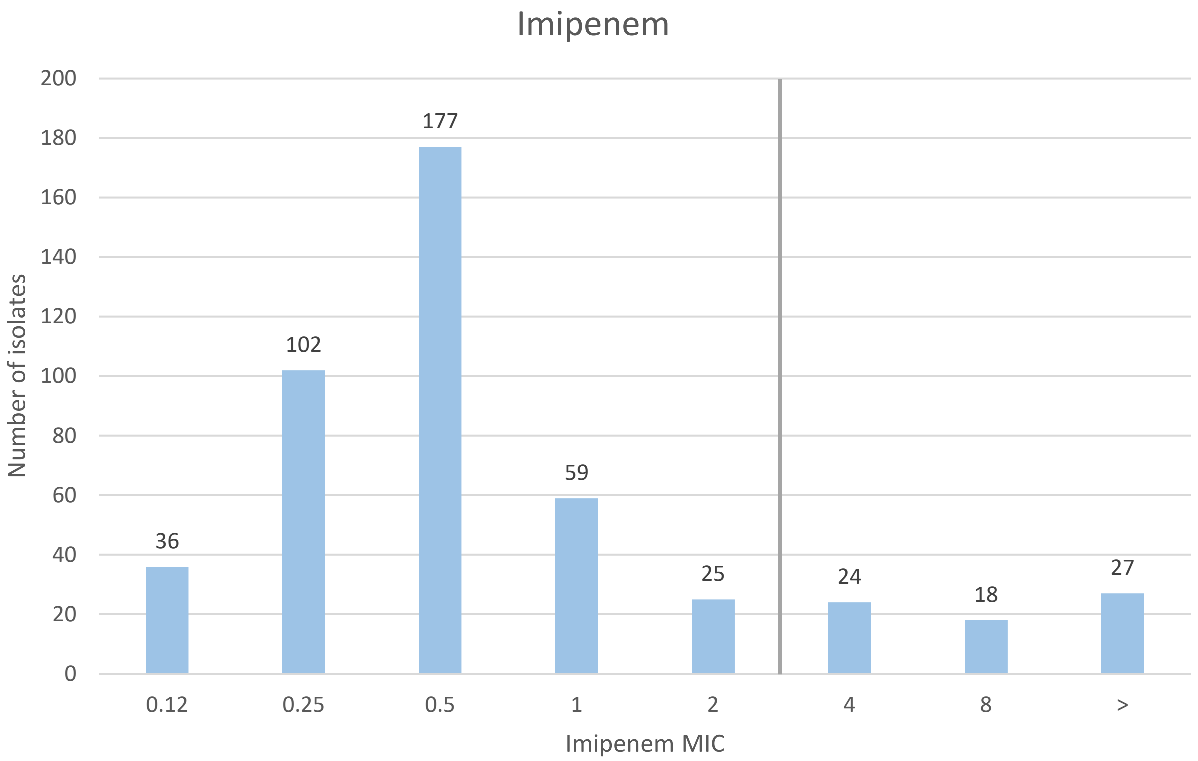

ECOFF Finder @ 97.5%: 2 ug/ml

Proposed MIC breakpoints		
**S**	**I**	**R**
<=2	4	>8

Levofloxacin MIC data (n = 467)

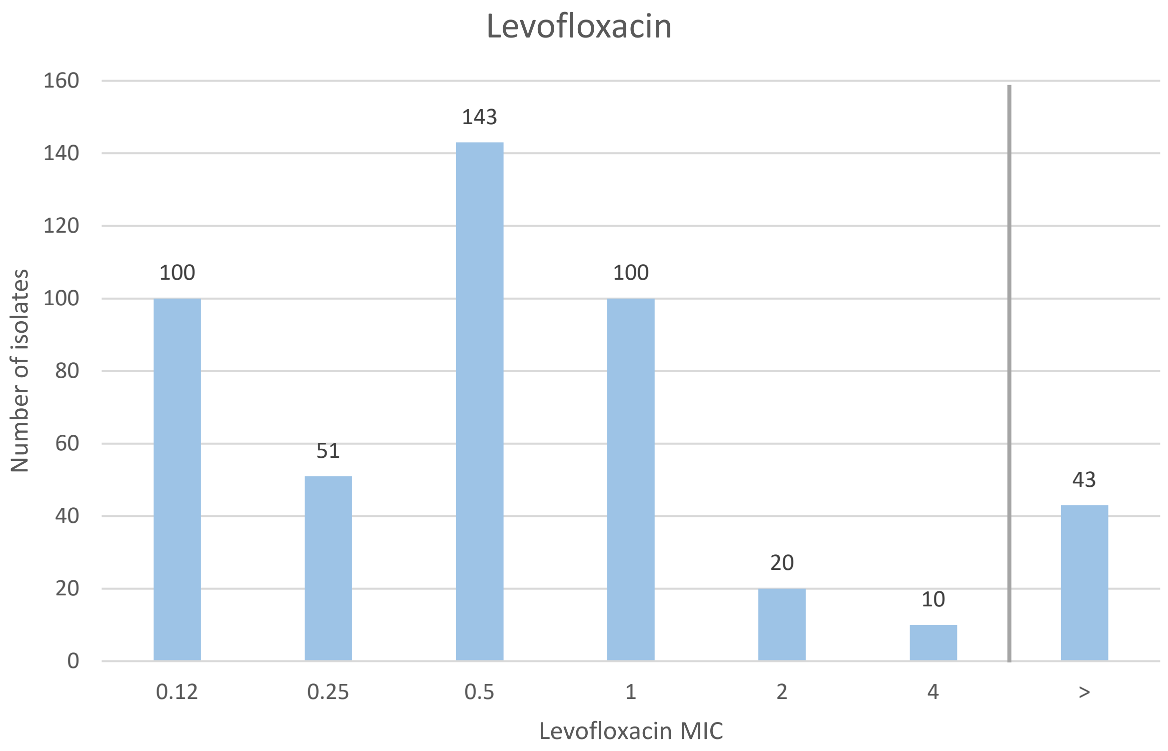

**ECOFF Finder @ 97.5%**: 4 ug/ml

**Proposed MIC breakpoints**		
**S**	**I**	**R**
<=1	2	>4

Meropenem MIC data (n = 467)

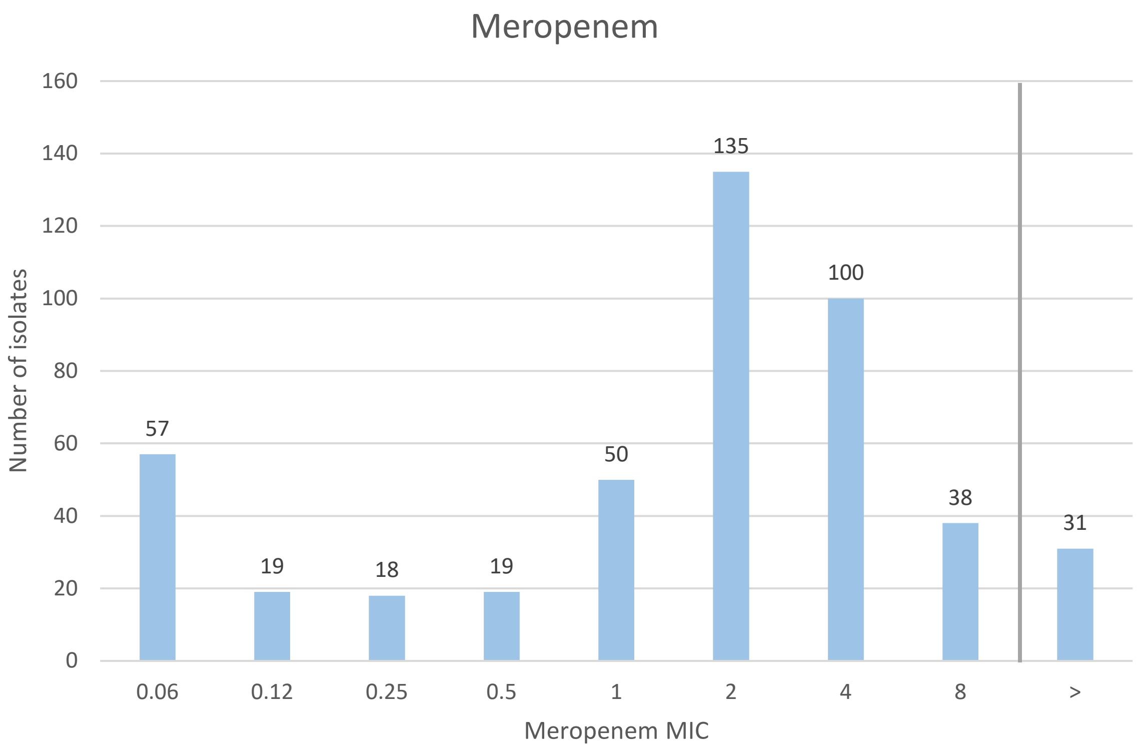

Proposed MIC breakpoints		
**S**	**I**	**R**
<=2	4	>8

Piperacillin-tazobactam MIC data (n = 469)

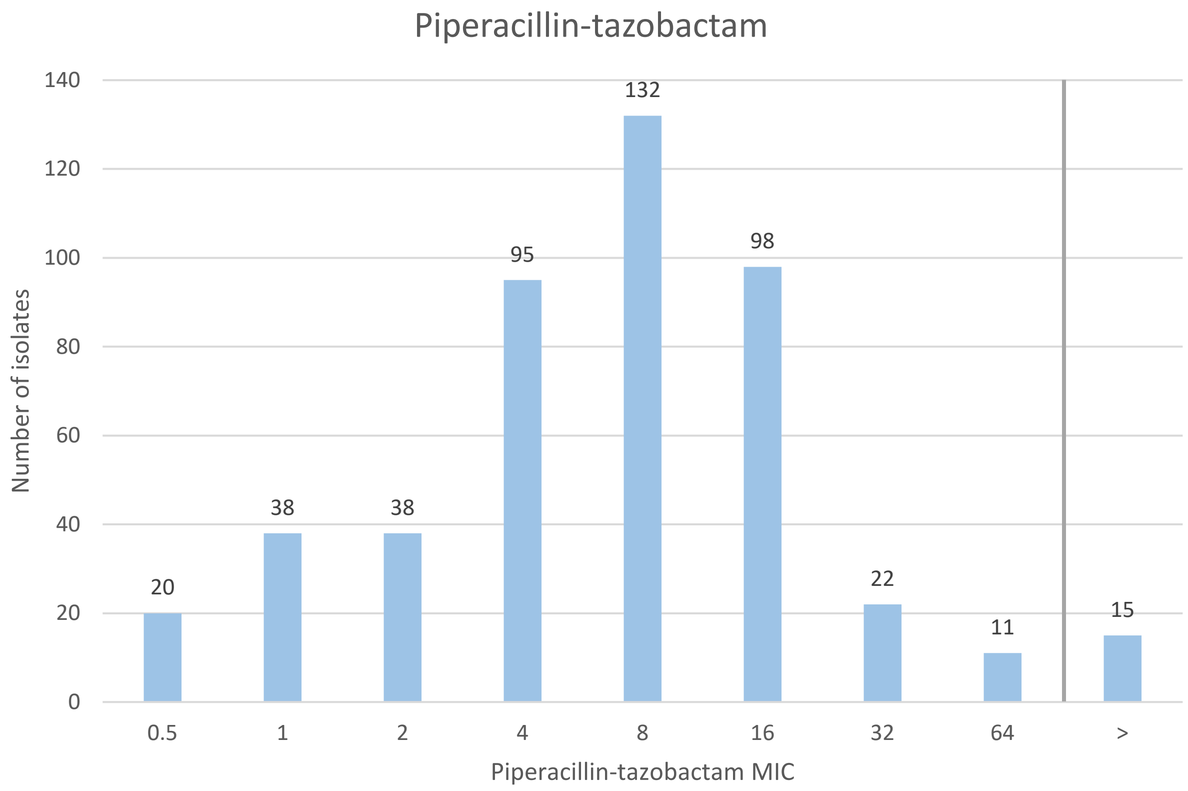

ECOFF Finder @ 97.5%: 64 ug/ml

Proposed MIC breakpoints		
**S**	**I**	**R**
<=16/4	32/4	>64/4

Tobramycin MIC data (n = 438)

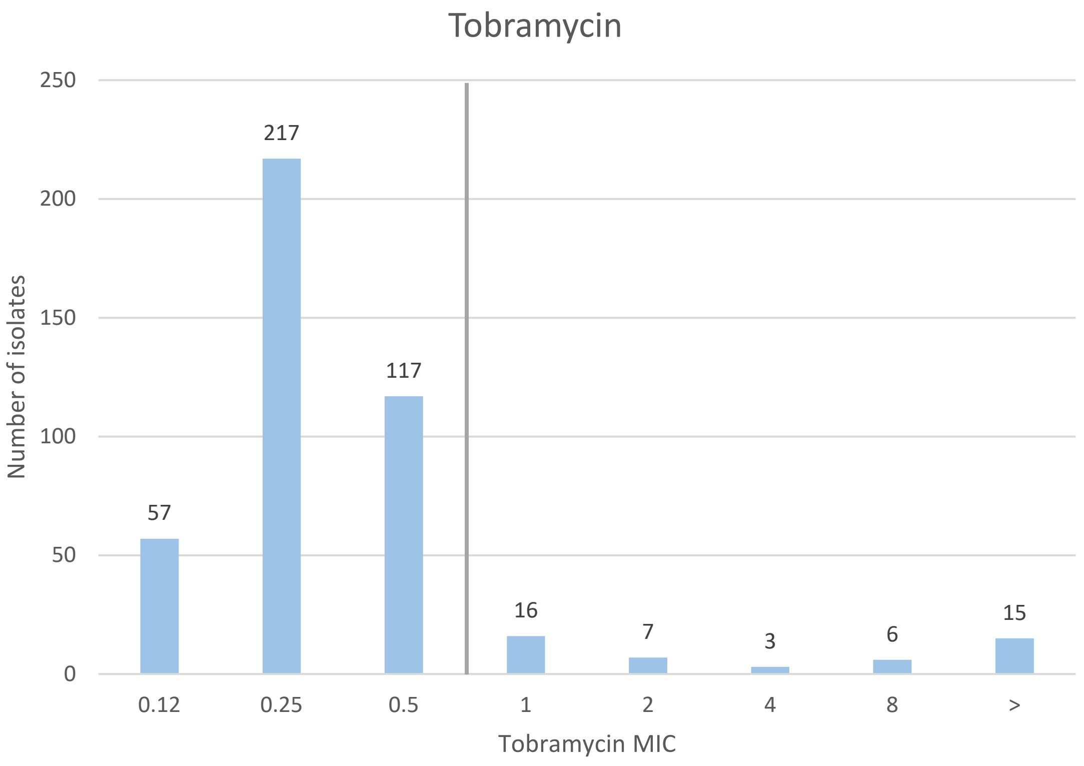

**ECOFF Finder @ 97.5%**: 0.5 ug/ml

**Proposed MIC breakpoints**		
**S**	**I**	**R**
<=1	2	>4

Trimethoprim-sulfamethoxazole MIC data (n = 468)

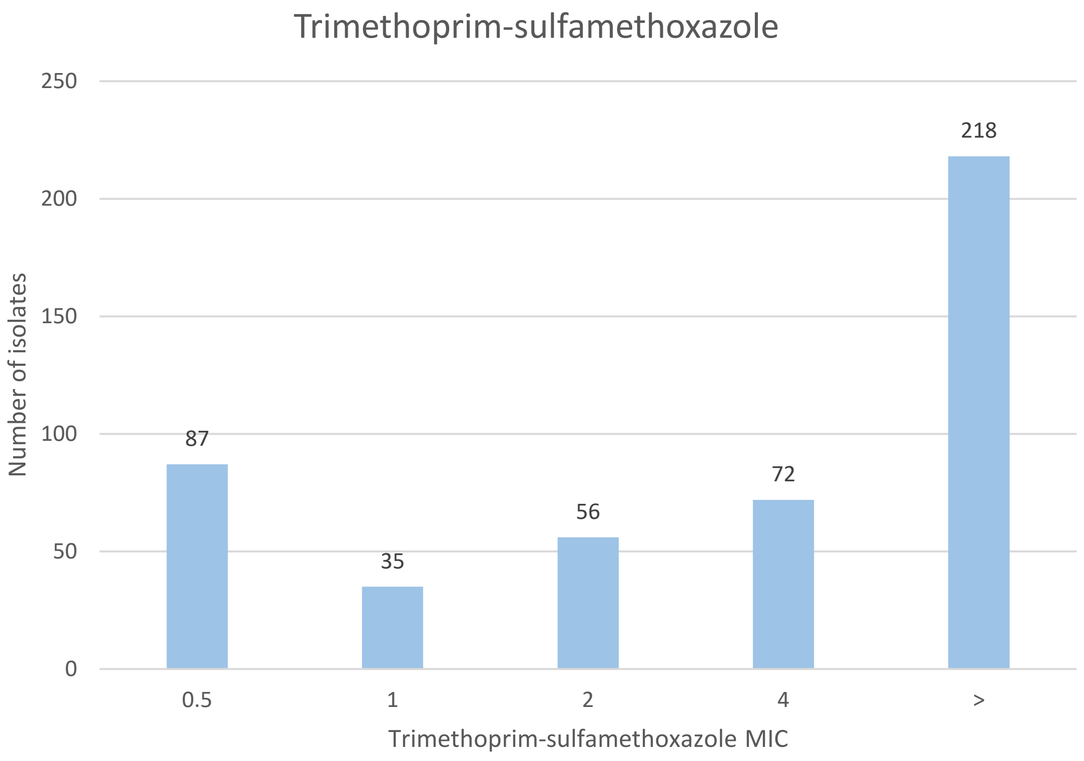

ECV could not be calculated

No proposed breakpoints

Piperacillin-tazobactam (TZP)

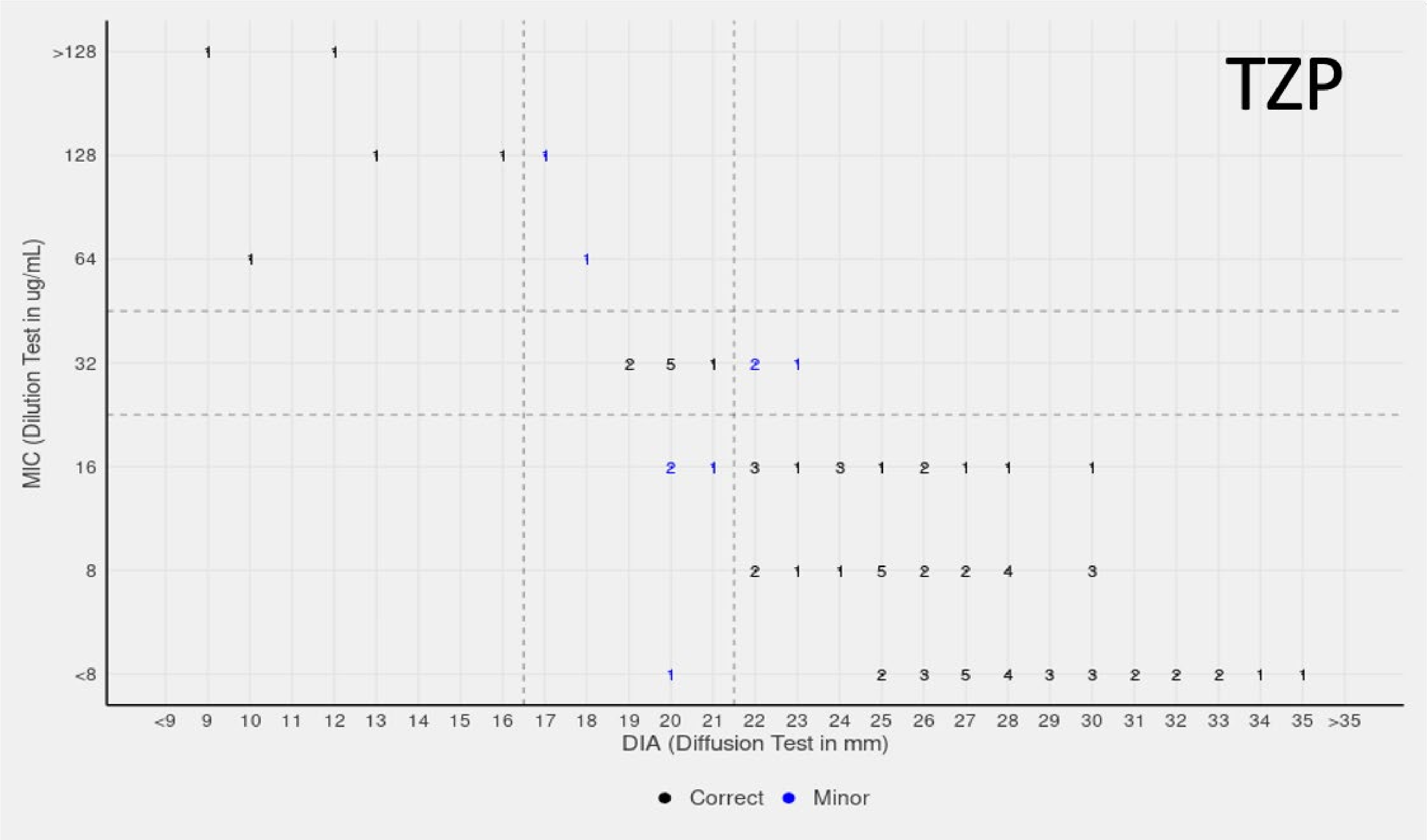

Ceftazidime (CAZ)

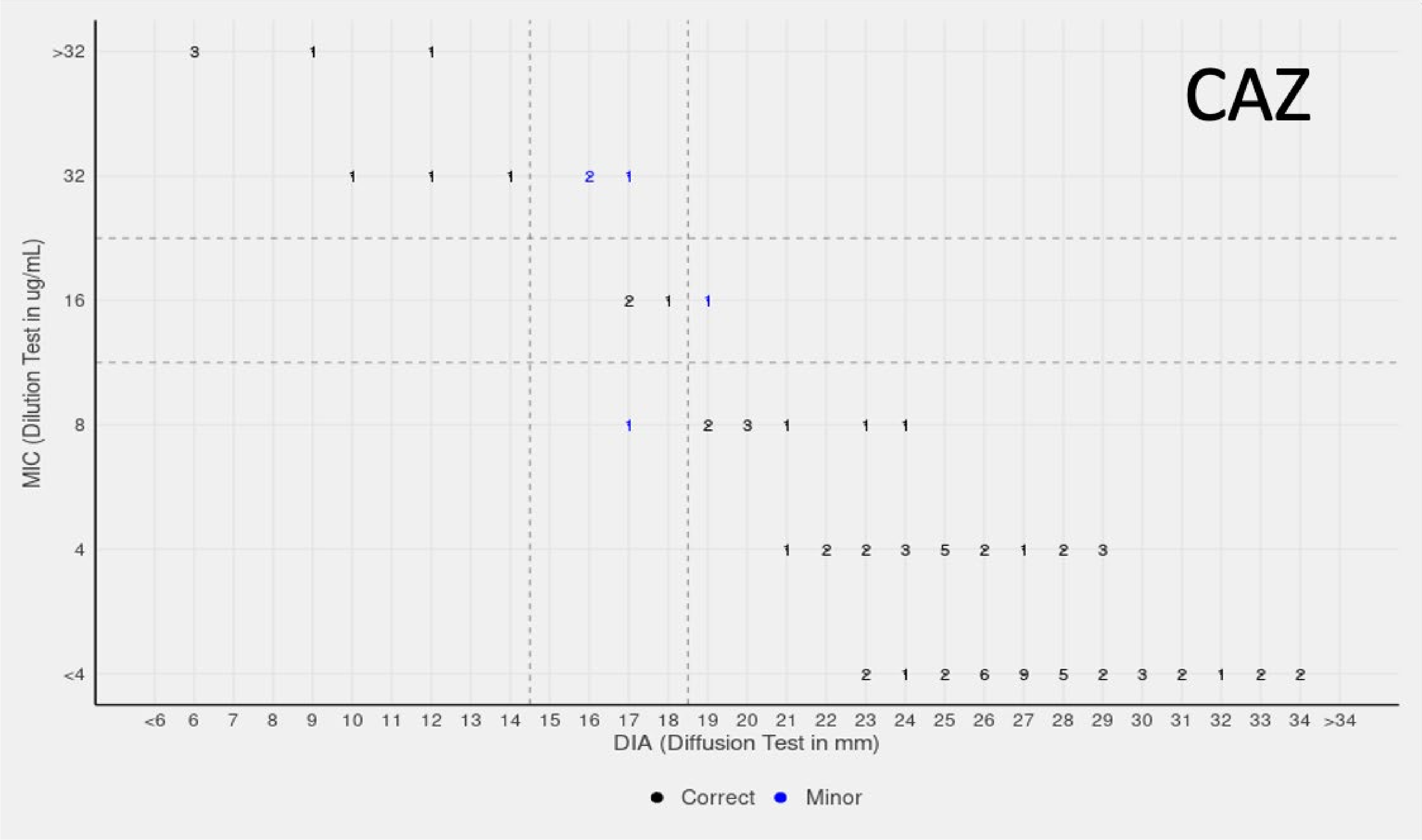

Cefepime (FEP)

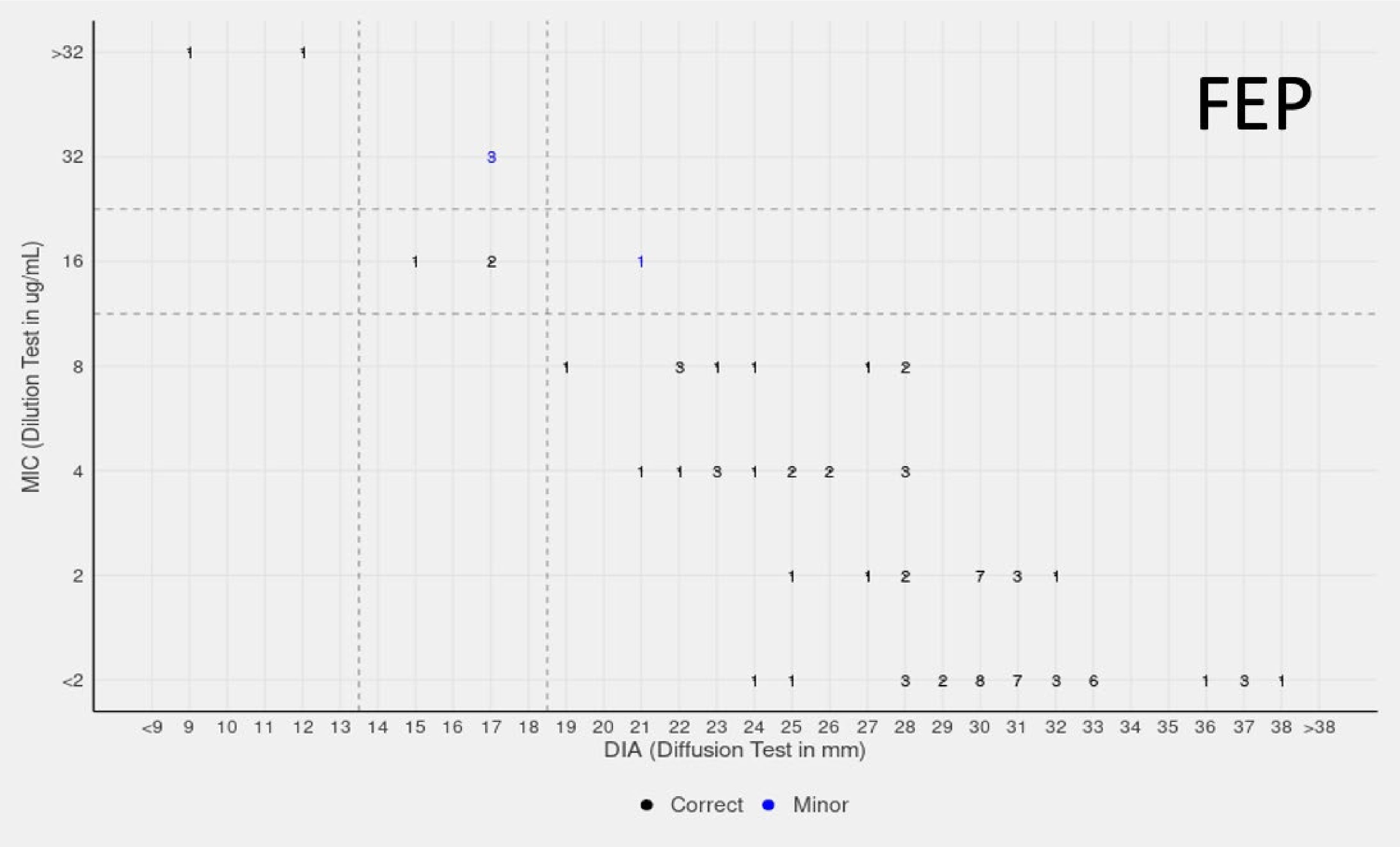

Imipenem (IMI)

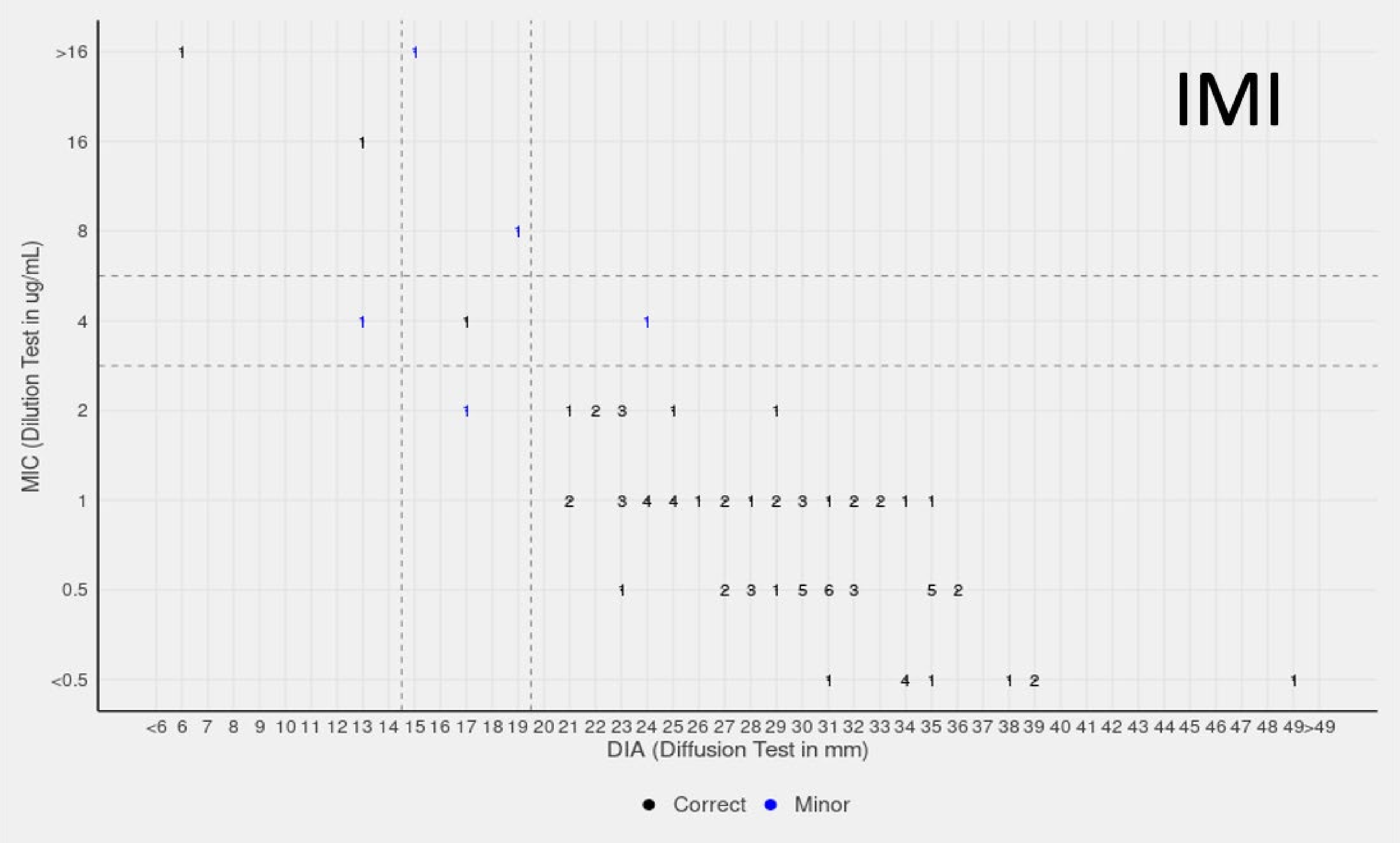

Meropenem (MER)

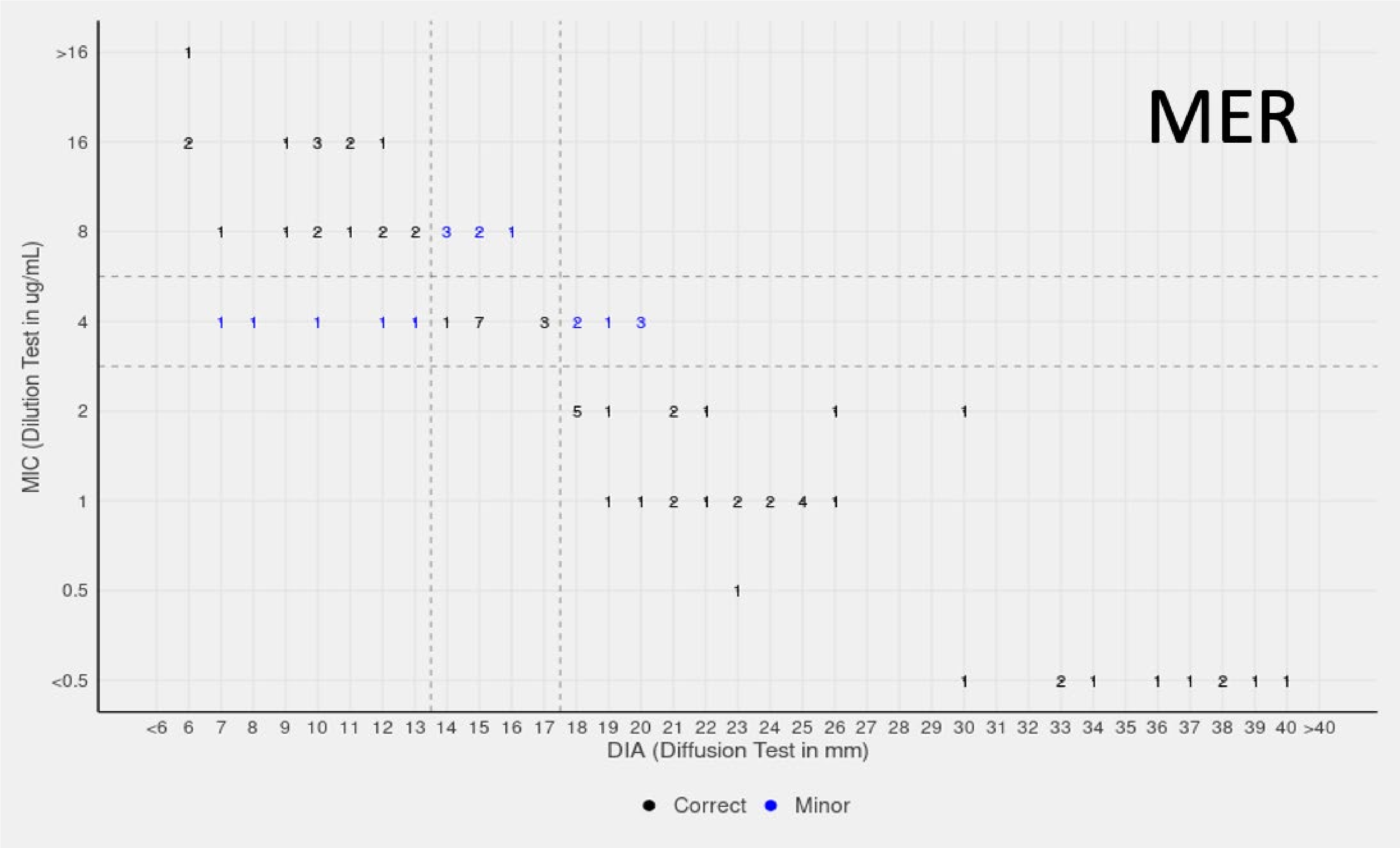

Tobramycin (TOB)

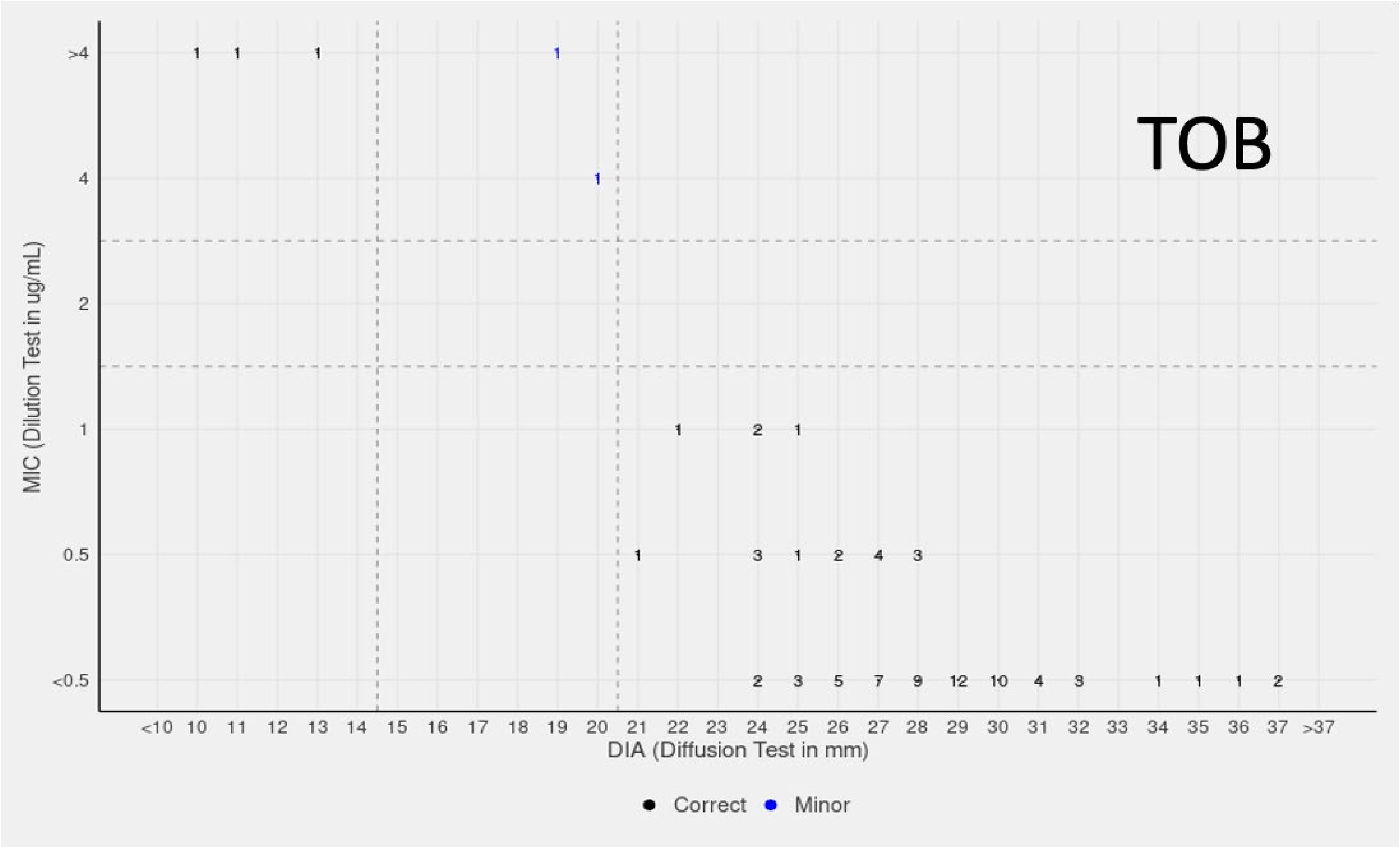

Ciprofloxacin (CIP)

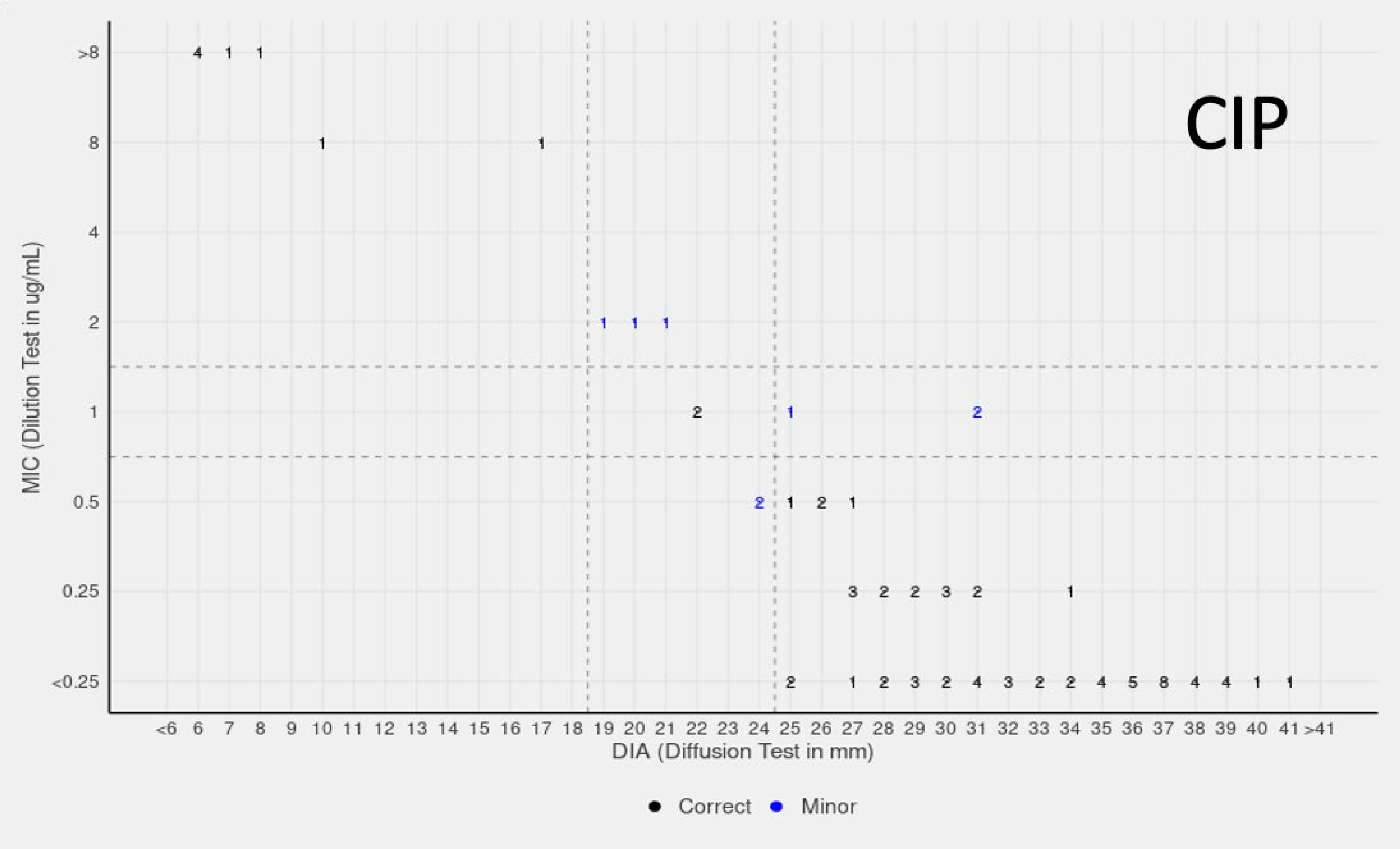

Levofloxacin (LEV)

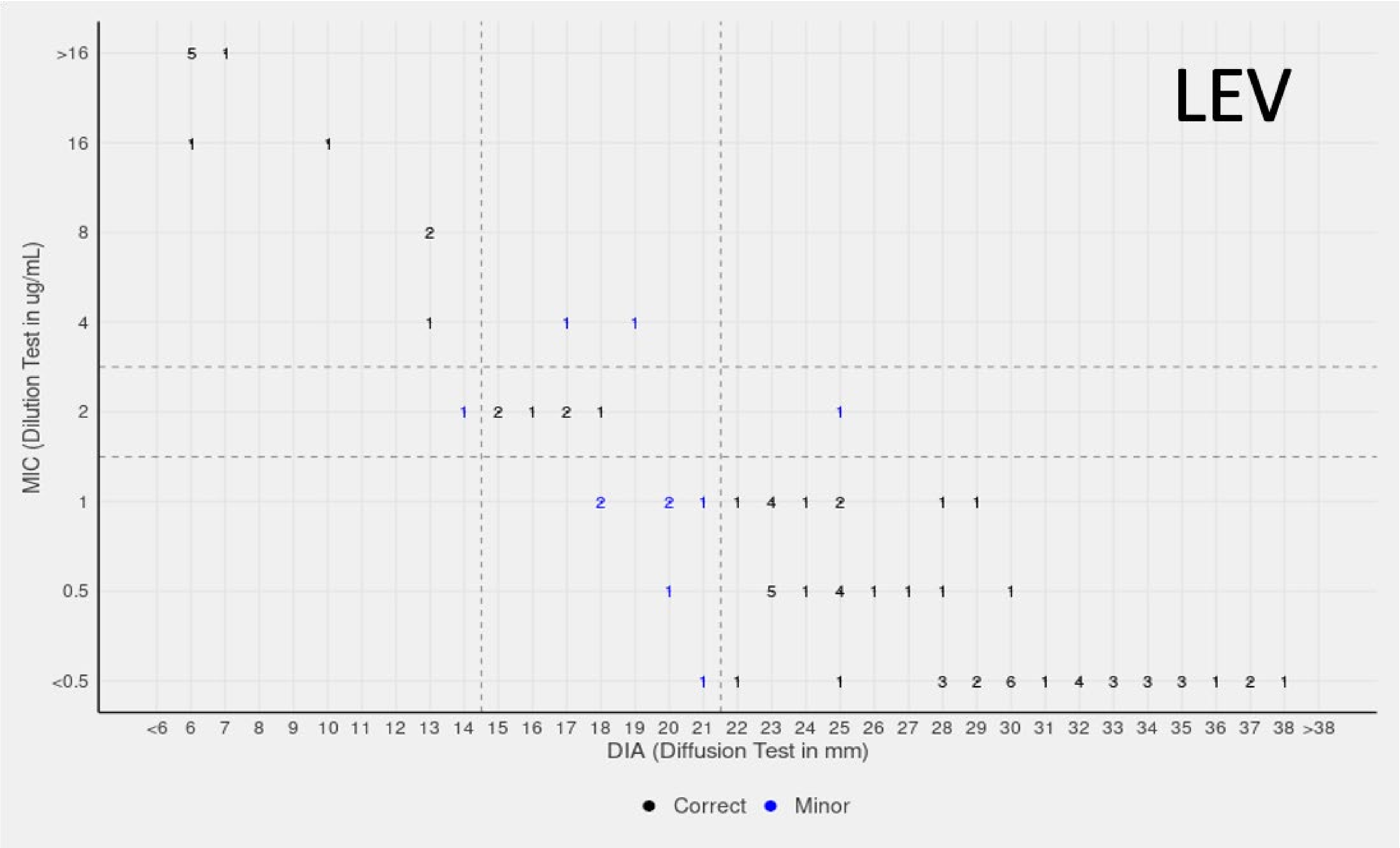

Supplement: Supplemental materials — M45 POPA histograms and scattergrams. [file jcm.00368-25-s0001.pdf]
